# Correction: Balkrishna et al. Application of Zebrafish Model in the Suppression of Drug-Induced Cardiac Hypertrophy by Traditional Indian Medicine Yogendra Ras. *Biomolecules* 2020, *10*, 600

**DOI:** 10.3390/biom14111341

**Published:** 2024-10-22

**Authors:** Acharya Balkrishna, Yashika Rustagi, Kunal Bhattacharya, Anurag Varshney

**Affiliations:** 1 Drug Discovery and Development Division, Patanjali Research Institute, Haridwar 249 401, India; 2 Department of Allied and Applied Sciences, University of Patanjali, Patanjali Yog Peeth, Haridwar 249 401, India

**Error in Figure**

In the original publication [1], there was a mistake in Figure 4 as published. Figure 4Biv and Figure 4Ciii were accidentally duplicated and represented the same heart gross pathology. The corrected Figure 4 appears below. 

**Figure 4:**
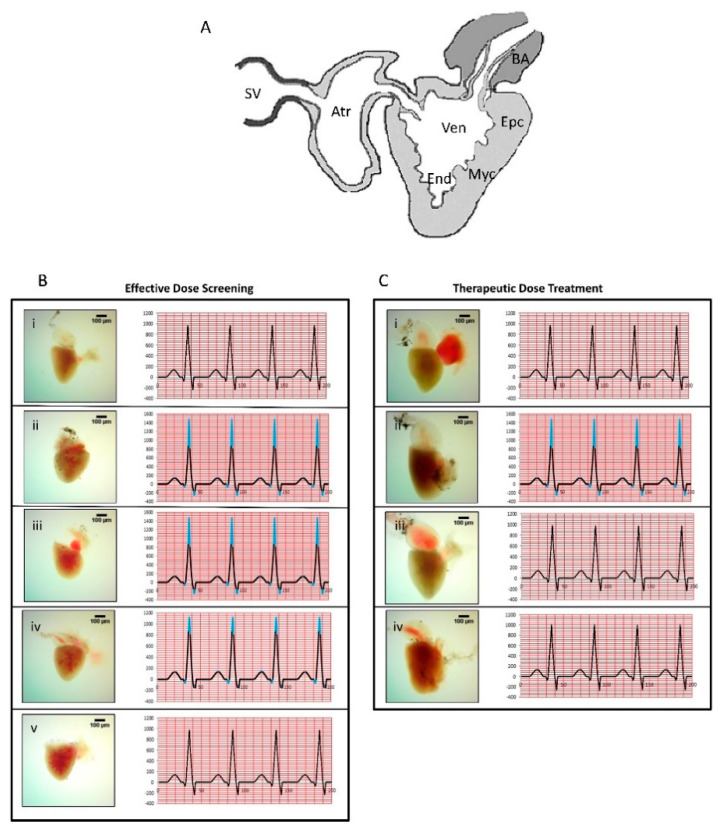
Electrocardiogram (ECG) analysis and whole heart histological analysis of erythromycin (ERY)-stimulated cardiac hypertrophy. (**A**) Schematic diagram of *Danio rerio* heart: sinus venosus = SV, atrium = Atr, ventricle = Ven, bulbus arteriosus = BA, epicardium = Epc, myocardium = Myc, endocardium = End (graphics adapted from Yacoub et al. [32]). (**B**) In the effective dose screening analysis, ECG and heart anatomy of the *D. rerio* were studied for (**i**) normal control; (**ii**) disease control (25 µg/Lt erythromycin (ERY)); (**iii**) low dose (0.6 µg/kg of YDR); (**iv**) medium dose (4 µg/kg of YDR); and (**v**) high dose (18 µg/kg of YDR). The results indicated induction of CH by ERY. No changes were detected in the CH parameters following the 7 days of effective dose treatment with YDR at the low and medium doses. Mild changes were observed at the highest tested dose of YDR. (**C**) In the therapeutic dose treatment study, *D. rerio* were grouped as (**i**) normal control; (**ii**) disease control (25 µg/Lt ERY); (**iii**) standard drug (4 µg/kg of verapamil); (**iv**) therapeutic dose of YDR (18 µg/kg of YDR). The results indicated that, following 14 days of treatment, both the verapamil and YDR successfully ameliorated the CH induced by ERY in *D. rerio*.

The correction made in the aforementioned figure does not affect the scientific findings or the conclusions presented in the manuscript. This correction was approved by the Academic Editor. The original publication has also been updated.

## References

[1] Balkrishna A., Rustagi Y., Bhattacharya K., Varshney A. (2020). Application of Zebrafish Model in the Suppression of Drug-Induced Cardiac Hypertrophy by Traditional Indian Medicine Yogendra Ras. Biomolecules.

